# Revisiting vitamin D status and supplementation for in-patients with intellectual and developmental disability in the North of England, UK

**DOI:** 10.1192/bjb.2021.55

**Published:** 2022-08

**Authors:** Bethany Dudley, Marcin Ostrowski, Vlad Ciausu, Chris Ince, Iain McKinnon

**Affiliations:** 1 Cumbria, Northumberland Tyne and Wear NHS Foundation Trust, Morpeth, UK; 2 Newcastle University, Newcastle upon Tyne, UK

**Keywords:** Autism spectrum disorders, forensic mental health services, intellectual disability, vitamin D, comorbidity

## Abstract

**Aims and method:**

To re-evaluate vitamin D testing and supplementation among in-patients with intellectual and developmental disability (IDD) and examine any correlates with physical health conditions, including COVID-19. Records of all in-patients between January 2019 and July 2020 (*n* = 78) were examined for 25-hydroxyvitamin D (25(OH)D) level, ward area, supplementation status, test seasonality, medication and health status.

**Results:**

The mean 25(OH)D level for supplemented (800 IU/day) patients was 75 nmol/L (s.d. = 20), compared with 40 nmol/L (s.d. = 19) in the non-supplemented group (*P* < 0.001). Thirty-eight percent of those who were in-patients during the first wave of the COVID-19 pandemic developed symptoms, but the small sample size could not establish vitamin D levels as a predictor of outcome.

**Clinical implications:**

Vitamin D (800 IU/day) supplementation is effective but the adequacy of the nationally recommended dose of 400 IU/day is unclear. Links to COVID-19 merit further research.

The active form of vitamin D (1,25-dihydroxyvitamin D) is a steroid hormone. It derives from a fat-soluble vitamin in two forms: vitamin D_2_ (ergocalciferol), which is of fungal origin, and vitamin D_3_ (cholecalciferol), which is of animal origin through dietary intake or produced in the skin from 7-dehydrocholesterol under the action of ultraviolet B radiation.^1^ The importance of vitamin D to bone health and the prevention of osteomalacia is well documented.^2^ There is also associated literature linking it to neuromuscular functioning and strength,^3^ as well as functional decline in older age.^4^ Vitamin D deficiency has also been linked to cardiovascular disease via observational studies.^5^ Insulin resistance^6^ and diabetic progression^7,8^ have also been linked to vitamin D status. Recent supplementation studies show promise with respect to some cancers.^9,10^ People with intellectual and developmental disability (IDD) are likely to be at greater risk of developing these chronic illnesses^11^ as well as being at increased risk of falls.^12^

A number of primary studies have highlighted vitamin D insufficiency and deficiency among groups of people with IDD,^13–15^ among institutionalised populations at a range of global latitudes^16–18^ and among those with comorbid epilepsy, in part due to enzyme-inducing medication.^19^ Studies among IDD populations have also investigated the risks of fractures and low bone mineral density,^20,21^ potentially as the result of vitamin D insufficiency. A population study demonstrated widespread low 25-hydroxyvitamin D (25(OH)D) levels in the UK population as a result of high latitude and low ultraviolet (UV) light levels, especially in the winter months.^22^ A more recent report by the Scientific Advisory Committee on Nutrition^23^ suggests lower levels still among institutionalised persons.

There has been recent renewed interest in the role of vitamin D. Its importance in immunity and respiratory health has been described, with actions of vitamin D proposed as underpinning the upregulation of the innate immune system and the reduction of pro-inflammatory cytokines.^24,25^ Studies have also investigated the impact on the severity of seasonal^26^ and epidemic influenza.^27^ Much of the new focus on vitamin D homeostasis and respiratory health has arisen during the SARS-CoV2 (COVID-19) pandemic. A small randomised controlled trial of immediate-release vitamin D versus placebo in Spain has showed promising results among COVID-19 in-patients,^28^ and a number of larger trials are now underway.^29,30^

In 2013–2014, we investigated the extent of vitamin D deficiency in the Northgate Hospital IDD in-patient services in Northumberland, UK.^31^ These services comprise medium secure, low secure and rehabilitation in-patient services for offenders with IDD, plus specialist tertiary in-patient autism services. We found widespread vitamin D deficiency and insufficiency, which led to the implementation of a supplementation protocol based on guidance^32^ provided by an expert National Health Service (NHS) prescribing guideline group on the basis of the evidence available at that time.^33^ However, since then, guidance on testing and dosing of vitamin D supplementation has changed locally^34^ and nationally,^35^ prompting us to reinvestigate our in-patient population.

The aim of the present study was to revisit the Northgate in-patient cohort to:

- assess the current extent of testing for 25(OH)D levels and the effectiveness of supplementation
- investigate whether patients who are started on supplementation are being retested to ensure adequate replacement and prevent over-treatment
- evaluate any correlates relating to physical health conditions that lead to increased risk, including any patterns relating to COVID-19 infection
- make recommendations for the current regime of supplementation and testing within in-patient IDD services and to consider whether other patients in secure settings should be offered a similar programme.

In this paper, we describe the results of this evaluation conducted at the end of 2020.

## Method

The study population comprised in-patients within any of the Northgate services at any point between 1 January 2019 and 31 July 2020. Wards sampled were the medium secure unit, low secure unit and hospital-based rehabilitation wards and the specialist autism in-patient services. The first three areas cater primarily for offenders with mild IDD, frequently with comorbid personality disorder or mental illness. The specialist autism service caters for people with complex autism spectrum conditions with a range of ability levels, from moderate ID to the normal intelligence range.

An anonymised database of patients was developed with data obtained from the hospital's electronic records. The study population was described based on ward location, level of security, age, ethnicity and medication, as well as testing and supplementation status. Information was gathered to indicate whether patients had been tested for vitamin D status during their hospital stay, and if so how long ago. We analysed patients’ 25(OH)D levels at their last test and whether patients were on supplementation at the time. For those taking supplementation at the time of the last test, the type, dose and duration of treatment were also recorded. In addition, we noted the prescription of psychotropic medication and enzyme-inducing medication.

We also noted how long ago it was since patients were last tested for 25(OH)D level and whether people started on supplementation were retested. The time since last test was taken as either time to 31 July 2020 if they were still an in-patient or the discharge date if discharged within the sampling window.

We performed a correlation to see whether there was an association between 25(OH)D level and length of time on treatment. In addition, we compared the groups with adequate and inadequate 25(OH)D levels for age, ethnicity, seasonality, ward location and psychotropic medication. Data on physical health risk factors, obesity and COVID-19 infection were also collected. The physical comorbidities of patients were described to evaluate whether there were any emerging patterns relating to COVID-19 infection.

Four authors (B.D., M.O., V.C. and I.M.) collected and analysed the data between October and December 2020. Data were collected on an MS Excel spreadsheet and analysed using IBM SPSS version 25 for Windows 10.

### Regulatory approvals

Ethical approval was not required for this service evaluation. It was registered with the Research and Development Department of Cumbria, Northumberland, Tyne and Wear NHS Foundation Trust in September 2020 (registration number SER-20-046).

## Results

### Patient sample

There were 67 in-patients in Northgate IDD services on 1 January 2019, with 11 further patients admitted up to the end of the sampling period on 31 July 2020, so the sample comprised 78 patients (19 of whom were discharged during that period). Ages were comparable across three of the four settings, except for an older group of patients in the hospital-based rehabilitation wards (one-way analysis of variance (ANOVA), d.f. = 3, *F* = 11.9, *P* < 0.001); the cohort is described in Table 1.

**Table 1:** Description of the cohort

Ward area	*n*	Age, years: mean (s.d.)	Gender		BAME	Medication			Season of test (*n* = 74)				Not tested	On supplementation at last test^a^	On supplementation on 1 Aug 2020 (or on discharge)
			M	F		Antipsychotics	Anticonvulsants	Enzyme inducing	Winter	Spring	Summer	Autumn			
MSU	25	33 (8)	25	0	1 (4%)	16 (64%)	8 (32%)	1	11 (44%)	4 (16%)	5 (20%)	5 (20%)	0	15/25 (60%)	24 (96%)
LSU	15	34 (10)	15	0	0	7 (47%)	3 (20%)	1	4 (29%)	3 (21%)	4 (29%)	3 (21%)	1	7/14 (50%)	14 (93%)
HBR	15	52 (18)	15	0	0	6	2	1	6 (40%)	4 (27%)	3 (20%)	2 (13%)	0	10/15 (67%)	14 (93%)
Autism	23	32 (8)	20	3	6 (26%)	21 (91%)	12 (52%)	0	5 (25%)	7 (35%)	3 (15%)	5 (25%)	3	15/20 (75%)	18 (78%)
Total	78	36 (13)	75	3	7 (9%)	50 (68%)	25 (34%)	3	26 (35%)	18 (24%)	15 (20%)	15 (20%)	4	47/74 (64%)	70 (90%)

BAME, Black, Asian and minority ethnic origin; M, male; F, female; MSU, medium secure unit; LSU, low secure unit; HBR, hospital-based rehabilitation; autism, specialist autism service.

a Note different denominator as not all patients were tested.

### Vitamin D testing during in-patient stay

Of the 78 patients in the sample, 74 had 25(OH)D levels tested at least once during their in-patient stay. Of these, the most recent test had been carried out within the past year for 19 patients, between 1 and 2 years for 20 patients, and over 2 years ago for the remaining 35 patients tested. Six of the latter group were last tested more than 5 years ago. Overall, 70 (90%) patients remained on vitamin D supplementation on the 31 July 2020 or on their date of discharge (which we will henceforth refer to as ‘day zero’).

Four patients had not been tested; review of their notes revealed several reasons, ranging from patient choice to clinical omission. One of the four patients was on vitamin D supplementation despite no record of serum testing.

The median time between the last test and day zero was 571 days. Many of the 23 patients who had insufficiency/deficiency detected at their last test, and who were subsequently prescribed supplements, had not been retested for substantial periods (median: 652 days; interquartile range: 826 days).

There was no difference in the time since last test between the group who were on (*n* = 69) compared with those who were not on (*n* = 5) supplements on day zero (Mann–Whitney *U* = 115, exact *P* = 0.22). Similarly, there was no difference in the time since last test between the group who had adequate 25(OH)D levels (*n* = 50) and the group with inadequate levels (*n* = 24) (*U* = 542, *P* = 0.50).

### Results of vitamin D testing

**Fig. 1:**
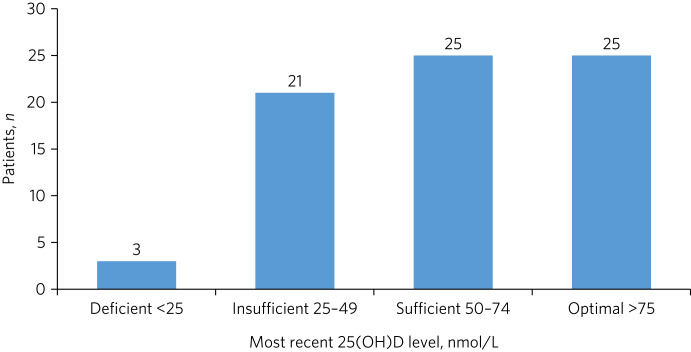
Vitamin D status of tested patients (*n* = 74). 25(OH)D, 25-hydroxyvitamin D.

The mean 25(OH)D level at the last test for 74 tested patients was 63 nmol/L (s.d. = 26). Values were normally distributed (Shapiro–Wilk: 0.98, *P* = 0.35) (Fig. 1).

The mean 25(OH)D level of the patients on supplementation at the time of the last test was 75 nmol/L (s.d. = 20). This was significantly higher than the mean for those not on supplements at the time (40 nmol/L, s.d. = 19, *t*-test 7.6, *P* < 0.001). Of the 47 who were taking supplements on the date of their last test, 3 (6%) were in the insufficient range (25–49 nmol/L); the remainder had levels ≥50 nmol/L. Forty (85%) were taking 800 IU of vitamin D, five were taking 400IU and two were taking 1600 IU. There was no difference in 25(OH)D levels between dosing groups (one-way ANOVA, d.f. = 2, *F* = 0.63, *P* = 0.54). Among the 27 who were not on supplementation when they were last tested, only 6 (22%) had 25(OH)D levels ≥50 nmol/L, with 18 (67%) in the ‘insufficient’ range and 3 (11%) having deficiency.

For those on treatment, there was no correlation between the length of time they were on treatment prior to the test and their 25(OH)D level (Pearson correlation: −0.15, *P* = 0.32).

We compared the adequate-levels group with the inadequate group. They were comparable for age, season of testing, ward location and whether they were taking antipsychotics or anticonvulsants (Table 2). All seven patients of Black or Asian minority ethnic origin were in the adequate group; all were on supplements at the time (five on 800 IU and two on 1600 IU). In addition, there were three patients (4%) on enzyme-inducing medication when tested – all were on supplements and all had 25(OH)D levels ≥50 nmol/L.

**Table 2:** Comparison of patients with adequate versus inadequate vitamin D levels^a^ (*n* = 74)

Group	Demographic		Season of latest test				Ward area				Psychotropic medication	
	Age, years: mean (s.d.)	BAME	Winter	Spring	Summer	Autumn	MSU	LSU	HBR	Autism	Antipsychotics	Anti-epileptics
Adequate (*n* = 50)	36 (14)	7 (14%)	21 (42%)	9 (18%)	9 (18%)	11 (22%)	15 (30%)	7 (14%)	12 (24%)	16 (32%)	32 (64%)	20 (41%)
Inadequate (*n* = 24)	38 (12)	0 (0%)	5 (21%)	9 (38%)	6 (25%)	4 (17%)	10 (42%)	7 (29%)	3 (13%)	4 (17%)	15 (63%)	5 (21%)
	*t* = 0.65 *P* = 0.52	Fisher's *P* = 0.09	χ^2^ = 5.22, d.f. = 3, *P* = 0.16				5.09, d.f. = 3, *P* = 0.17				χ^2^ = 0.02, d.f. = 1, *P* = 0.90	χ^2^ = 2.66, d.f. = 1, *P* = 0.10

a. Adequate levels: 25-hydroxyvitamin D (25(OH)D) level ≥50 nmol/L; inadequate levels: 25(OH)D level <50 nmol/L.

BAME, Black, Asian and minority ethnic origin; MSU, medium secure unit; LSU, low secure unit; HBR, hospital-based rehabilitation; autism, specialist autism service.

### Patients tested on admission

Within the cohort, 12 patients’ tests were within 28 days of admission to hospital. Four were on supplements on admission (three from other hospitals and one from the community); all had adequate 25(OH)D levels. The remaining eight (two from prison and six from other hospitals) were not on supplements on admission and all had inadequate levels.

### Physical health and COVID-19 infection

We gathered data on chronic physical health conditions and body mass index (BMI), as well as whether patients had tested positive or had symptoms of COVID-19 during the first wave in 2020, along with the severity of infection (Table 3). Most chronic health problems were found in the hospital-based rehabilitation group, who were older than the rest of the cohort. Only 14% of the patients were in the normal BMI range, with 59% classed as clinically obese. Of the 64 patients who were in-patients during the first wave of COVID-19 (February to July 2020), 17 (27%) tested positive for COVID-19, with a further 7 (11%) showing typical symptoms but having a negative polymerase chain reaction (PCR) test. Only one patient required a short transfer to an acute hospital and no patients died. There was no difference in the proportion of patients taking vitamin D supplements between the three COVID-status groups: (a) no symptoms: 36/40 (90%); (b) symptomatic with a positive test: 15/17 (88%); (c) symptomatic with a negative test: 7/7 (100%) (χ^2^ = 0.86, d.f. = 2, *P*=0.65).

**Table 3:** Summary of physical comorbidities, body mass index (BMI) and COVID-19 status

Ward area	*n*	Physical health								BMI, kg/m^2^				COVID-19		
		Type 2 diabetes mellitus	CKD	CVD	Hyper-tension	Asthma	Epilepsy	Cerebral palsy	Cancer (historical)	<25	25–30	>30	Refused weighing	In-patient during first wave	Positive PCR test (hospitalised)	Symptoms but no positive test
MSU	25	4	0		1	5	4	0	0	6	7	12	0	21	9 (0)	1
LSU	15	1	1	1 CM	1	4	1	0	0	1	3	11	0	12	4 (1)	1
HBR	15	2	0	1 CAD	8	2	2	1	0 (1)	1	5	9	0	12	0 (0)	1
Autism	23	3	0	1 CAD	3	1	5	0	0	3	5	14	1	19	4 (0)	4
Total	78	10 (13%)	1 (1%)	3 (4%)	13 (17%)	12 (15%)	12 (15%)	1 (1%)	0 (1) (1%)	11 (14%)	20 (26%)	46 (60%)	1	64 (82%)	17 (1) (27%)	7 (11%)

CKD, chronic kidney disease; CVD, cardiovascular disease; PCR, polymerase chain reaction; MSU, medium secure unit; LSU, low secure unit; HBR, hospital-based rehabilitation; autism, specialist autism service; CM, cardiomyopathy; CAD, coronary artery disease.

## Discussion

This paper describes a follow-up to our 2013–2014 data published in 2018.^31^ The aim was to assess the legacy of a programme of vitamin D testing and supplementation implemented across our in-patient services in 2013.^32^

Our findings show that clinicians continue to offer vitamin D supplementation for in-patients, at a dose of 800 IU (20 μg) per day. The mean 25(OH)D levels we observed were higher for those on supplements compared with our 2013 baseline data, whereas patients not on supplementation now had levels akin to those found in our previous paper (35 nmol/L in 2013 versus 40 nmol/L now). Most patients on supplementation have levels in the adequate (≥50 nmol/L) range as defined by our local testing laboratories. All newly admitted patients arriving on vitamin D supplementation had adequate levels, regardless of admission source, compared with those arriving un-supplemented. Most admissions to Northgate are interhospital transfers, hence it is not possible to comment definitively on community 25(OH)D levels from these data. This requires further research, although currently available evidence from community studies of people with IDD^15^ and the wider population^22,23^ suggest that people anywhere in the UK are at risk, with greater risks in the North of England and Scotland. Studies have shown that latitudes more than 52° north of the equator, as Northgate Hospital is, are not compatible with cutaneous synthesis of vitamin D_3_ between October and March.^36^ Studies have also shown that hospital in-patients from warmer latitudes with higher UV indices also have a propensity for vitamin D deficiency.^18^

### Testing and dosing regimes

There remains a question of when to test and retest, if applicable. Current UK guidance does not recommend routine testing for vitamin D deficiency even in ‘high-risk’ patients such as ours, who are asymptomatic, instead recommending that all UK residents should take a daily supplement of 400 IU.^35^ Our original programme of vitamin D replacement consisted of prescription of 800 IU per day for people with insufficient levels, with initial loading treatment in the case of deficiency (levels <25 nmol/L). This dosing regimen remains widely practised and our data show that it leads to adequate replacement; it is not clear from our results whether dosing at 400 IU per day would have a similar beneficial effect. However, our data show that patients are at risk of ongoing insufficiency or deficiency while in secure hospitals. If not tested at baseline, we would advocate the testing of patients who start vitamin D supplements to ensure adequate replacement. There is a theoretical risk of hypercalcaemia, leading to renal stones or renal failure, although studies have reported safety data up to an upper limit of 10 000 IU per day.^37,38^ It is notable that these international data support supplementation well above UK recommended levels.

### Capacity to consent

Many patients within the secure cohort have mild intellectual disability and many are able to give informed consent for testing and supplementation. This is more of a challenge where patients lack capacity and best interests decisions are needed. Some of the patients within our autism services have more severe cognitive and communication problems, although conversely, more able patients with autism spectrum conditions may view medication with suspicion, and therefore steps to ensure an appropriate level of patient education using visual aids may be needed. Some patients may also be unwilling to take vitamin D supplementation without evidence of inadequate levels. Consequently, an individualised approach will be required.

### COVID-19 and other factors

The relevance of COVID-19 is twofold. First, the effects of restriction on movement may have further deleterious effects on cutaneous production of vitamin D, especially in the summer months, compounding the pre-existing risks of institutionalised living and boosting the case for supplementation (either in hospital or in the community). Second, there is growing evidence for the role of vitamin D homeostasis in effective immune and inflammatory responses to respiratory infections, including COVID-19,^39,40^ as well as being of potential benefit for a range of health problems.^41^ Our data show that our in-patient population have a range of comorbid physical health conditions. Almost all of our patients who had COVID-19 infection during the first wave in 2020 had mild infections, despite a range of physical comorbidities. However, it is not possible to make any inference from our data about the link between vitamin D status or supplementation and COVID-19 infection.

There are additional considerations, such as the impact of the general nutritional status of our in-patients. Many are overweight or obese, which affects vitamin D absorption by fatty tissues and has a theoretical effect on bioavailability. We did not study vitamin D-containing diets or foodstuffs among our patients; this is an area for future research.

### Implications

We do not currently routinely offer supplementation to patients within our sister general adult (non-IDD) forensic services. A study published from The State Hospital in North Lanarkshire, Scotland, which is at a similar latitude to Northgate Hospital, confirmed substantial vitamin D deficiency among patients with a range of psychiatric conditions in secure care.^42^ It is likely, then, that psychiatric patients in longer-term in-patient settings should be considered for supplementation and assessment of the adequacy of 25(OH)D levels. There is currently a small but growing body of evidence investigating links between vitamin D homeostasis and mental health problems, including autism spectrum conditions,^43–45^ attention-deficit hyperactivity disorder^46,47^ and depressive disorders.^48,49^

### Limitations

This study has a number of limitations. For some of the cohort, vitamin D levels were tested a relatively long time ago, which may affect the validity of the results. Furthermore, the number of patients in the study was small and therefore we were not able to see any significant findings with respect to COVID-19 infection. Future research should consider multicentre longitudinal cohorts of patients to attempt to assess a range of health outcomes.

## Data Availability

The data that support the findings of this study are available from the corresponding author on reasonable request.

## About the authors

**Bethany Dudley** is a core psychiatry trainee with Cumbria, Northumberland, Tyne and Wear NHS Foundation Trust, Morpeth, UK. **Marcin Ostrowski** is an associate specialist with Cumbria, Northumberland, Tyne and Wear NHS Foundation Trust, Morpeth, UK. **Vlad Ciausu** is a specialty doctor with Cumbria, Northumberland, Tyne and Wear NHS Foundation Trust, Morpeth, UK. **Chris Ince** is a consultant psychiatrist with Cumbria, Northumberland, Tyne and Wear NHS Foundation Trust, Morpeth, UK. **Iain McKinnon** is a consultant psychiatrist with Cumbria, Northumberland, Tyne and Wear NHS Foundation Trust, Morpeth, and an honorary senior clinical lecturer in the Faculty of Medical Sciences at Newcastle University, Newcastle upon Tyne, UK.
